# Retraction Note: The circular RNA circfarsa sponges microRNA-330-5p in tumor cells with bladder cancer phenotype

**DOI:** 10.1186/s12885-025-15198-2

**Published:** 2025-10-21

**Authors:** Chen Fang, Xin Huang, Jun Dai, Wei He, Le Xu, Fukang Sun

**Affiliations:** Department of Urology, Ruijin Hospital, Shanghai Jiao Tong University School of Medicine, No. 197, Ruijing 2nd Road, Shanghai, 200025 China

**Retraction Note: BMC Cancer 22, 373 (2022)**

**https://doi.org/10.1186/s12885-022-09467-7**

The Editors have retracted this article. A part of the right side panel of Fig. 6G appears to partially overlap with the BXPC-3 RAS + HDAC2-1 panel of Fig. 4E in a previously published article by different authors [1]. There also appears to be overlap between Fig. 6G sh-circFARSA + NC inhibitor panel of this paper and Fig. 5E panel SiHa miR-873-5p mimics in [2]. There also appears to be overlap of Fig. 6G panel for sh-circFARSA + miR-330 inhibitor, and Fig. 6H, panel sh-circFARSA + miR-330 inhibitor and HeLa si-NC panel in Fig. 7D in [2].

The Editors therefore no longer have confidence in the results and conclusions reported in this article.

## References

[1] Li M, Song SW, Ge Y, Jin JY, Li XY, Tan XD. The Ras-ERK signaling pathway regulates acetylated activating transcription factor 2 via p300 in pancreatic cancer cells. Ann Transl Med. 2020;8(19):1234. 10.21037/atm-20-5880. 33178766 10.21037/atm-20-5880 PMC7607129

[2] Zhang S, Chen Z, Sun J, et al. RETRACTED ARTICLE: circrna hsa_circRNA_0000069 promotes the proliferation, migration and invasion of cervical cancer through miR-873-5p/TUSC3 axis. Cancer Cell Int. 2020;20:287. 10.1186/s12935-020-01387-5. 32655319 10.1186/s12935-020-01387-5 PMC7339483

